# Supplementary figures and images for: Towards increased accuracy and reproducibility in SARS-CoV-2 next generation sequence analysis for public health surveillance
Source: bioRxiv. 2022 Nov 3:2022.11.03.515010. Preprint. [Version 1] doi: 10.1101/2022.11.03.515010 (PMC9645426; doi:10.1101/2022.11.03.515010)

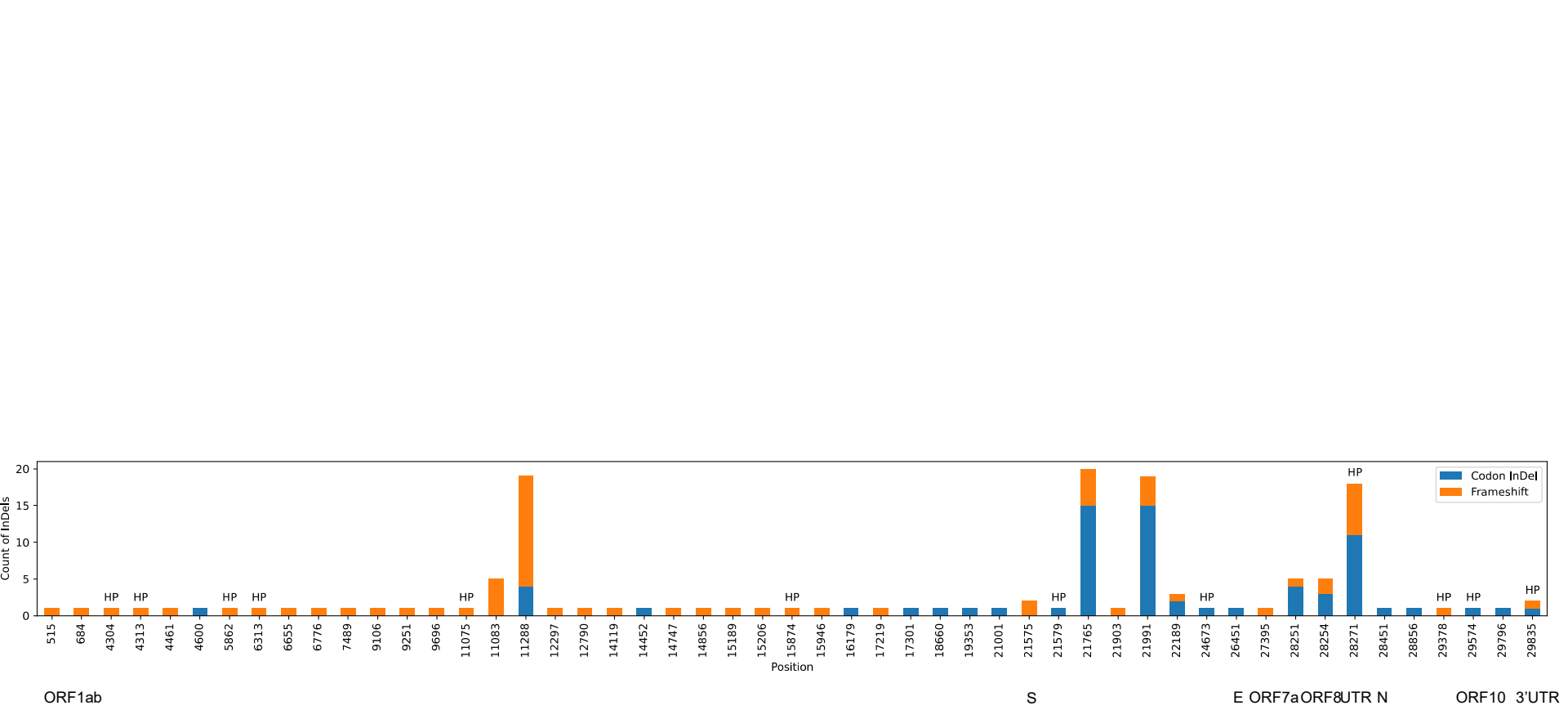

ORF1ab

S

E ORF7aORF8UTR N

ORF10 3'UTR

Supplement: Supplement 2 — Supplemental Figure 1. Indel Calls across the length of the SARS-CoV-2 Genome. In-frame calls are indicated in blue, frameshifting calls are in orange. Calls in homopolymer regions are indicated by “HM.” Many of the calls are made in homopolymer regions, those earlier in the genome are more likely to be frameshifting, and only a few positions have InDels called many times across the dataset considered. [file media-2.pdf]

A

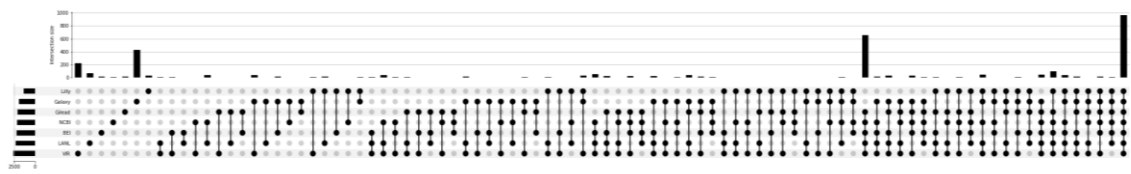

B

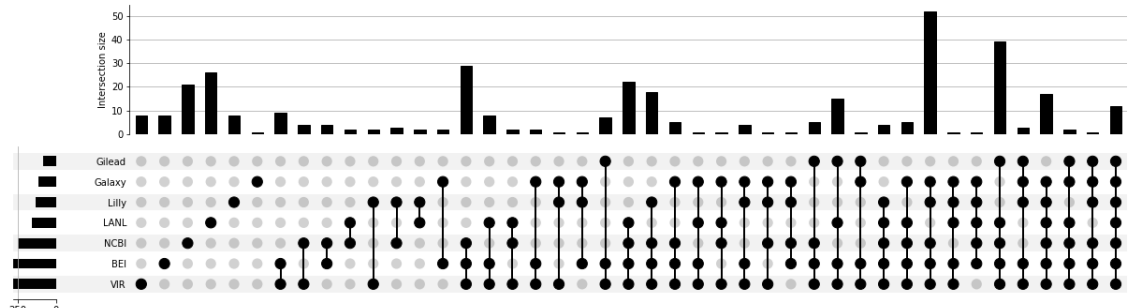

C

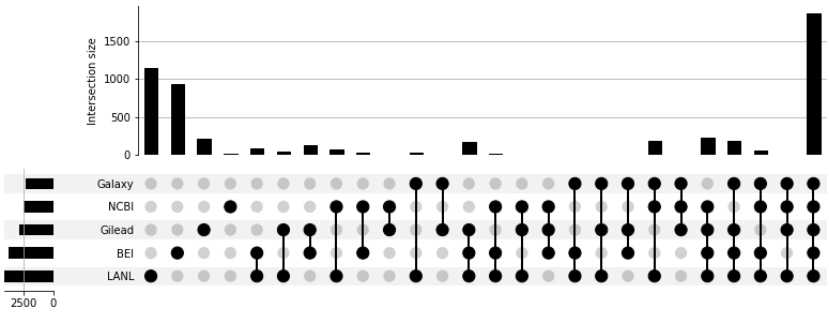

D

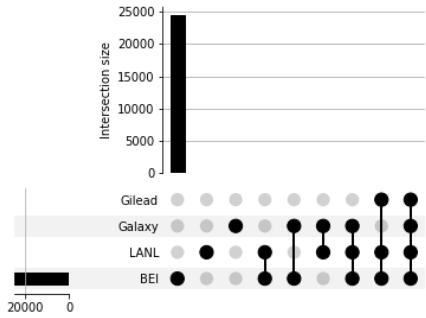

E

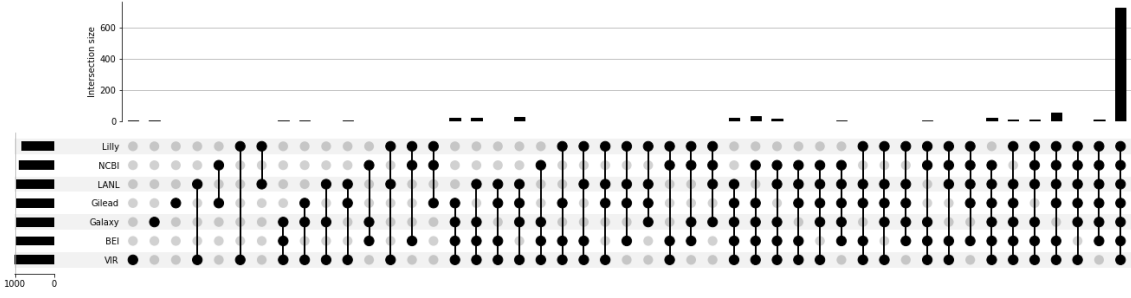

G

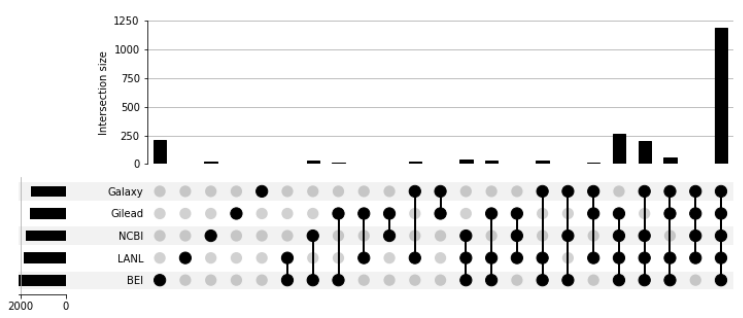

F

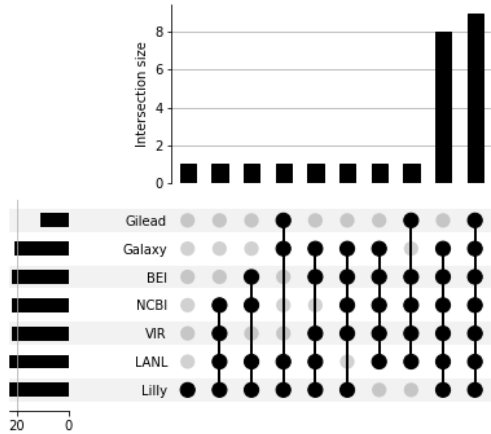

H

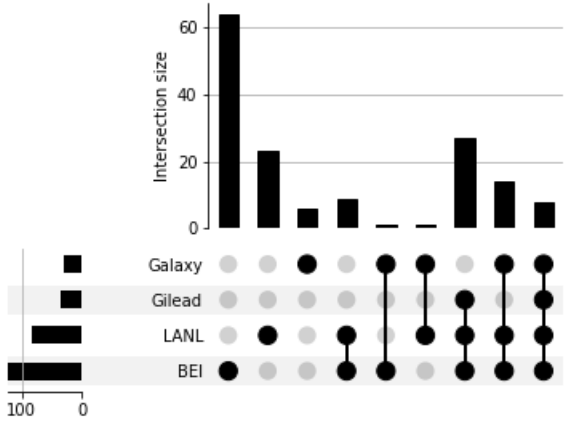

Supplement: Supplement 3 — Supplemental Figure 2. Agreement across pipelines with and without recommended parameters, recent dataset. A+B+C+D) Agreement across pipelines without recommended parameters. E+F+G+H) Agreement across pipelines with recommended parameters. A+E) Agreement on Illumina SNP calls. B+F) Agreement on Illumina InDel calls. C+G) Agreement on Oxford Nanopore (ONT) SNP calls. D+H) Agreement on ONT InDel Calls. For each figure, the bars indicate the number of variants called by the groups indicated by filled circles below, across the whole dataset. [file media-3.pdf]

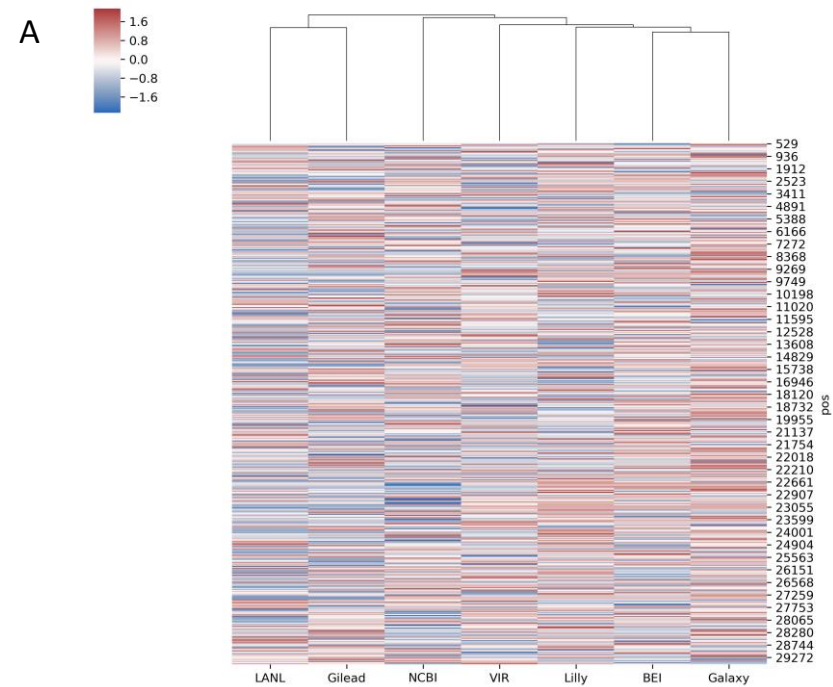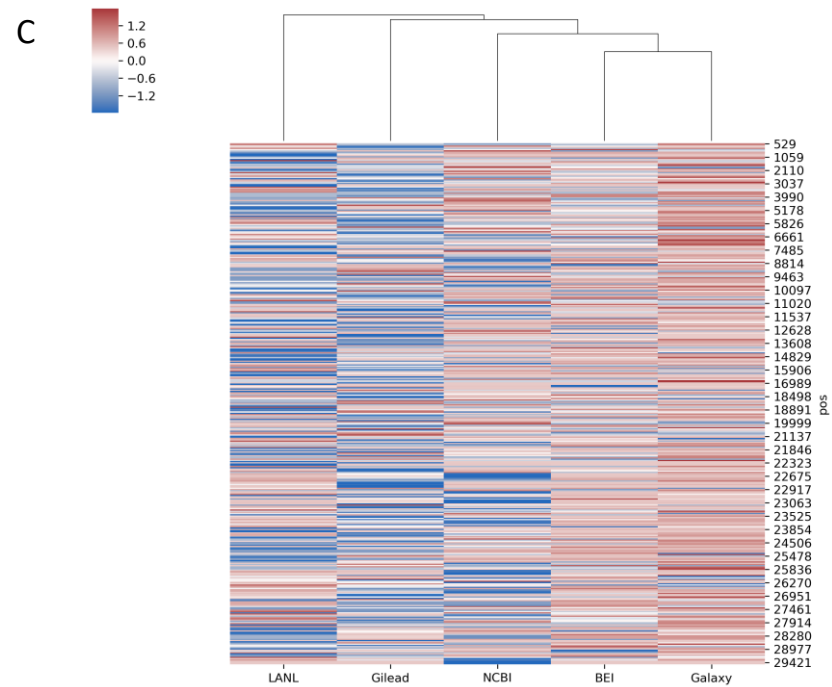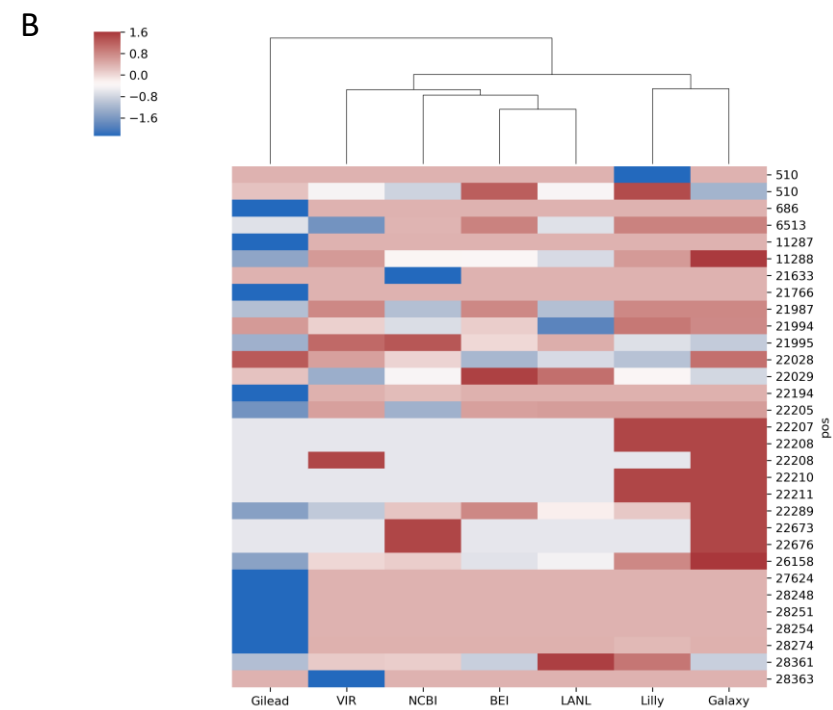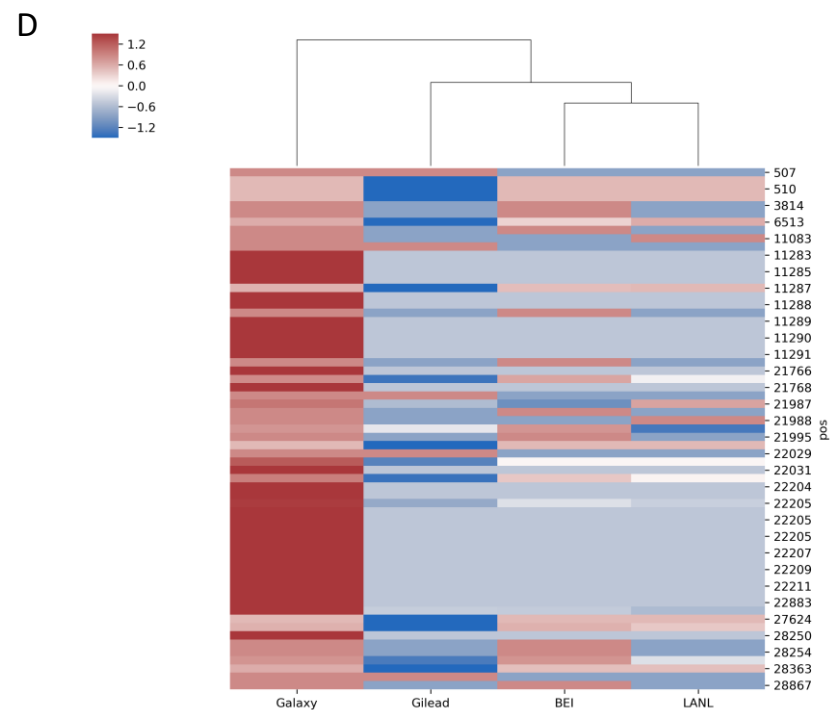

Supplement: Supplement 4 — Supplemental Figure 3. Difference in variant call frequencies across the length of the reference genome for each pipeline. A+B) Illumina platform data. C+D) ONT platform data. A+C) SNP calls. B+D) InDel Calls. For each pipeline, each row indicates a genomic position at which any pipeline called a variant. The color map indicates the difference in the frequency of the calls at that position, across the whole dataset, compared to the average frequency of calls made by all groups. [file media-4.pdf]

A

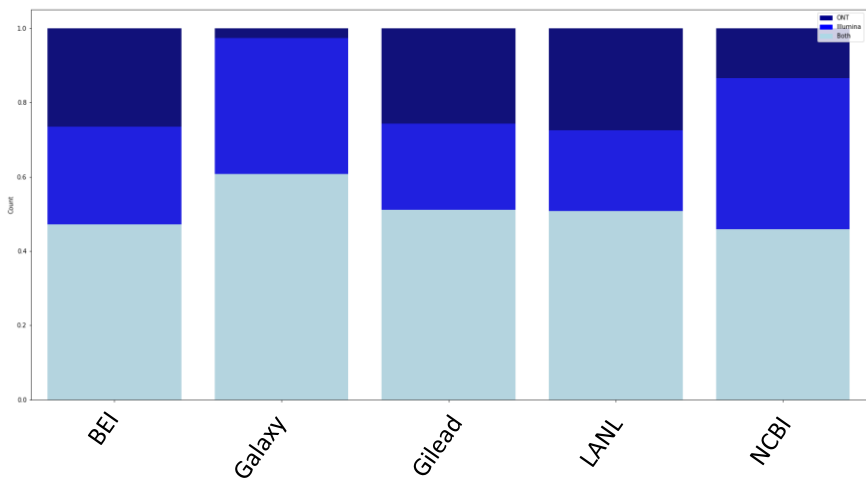

C

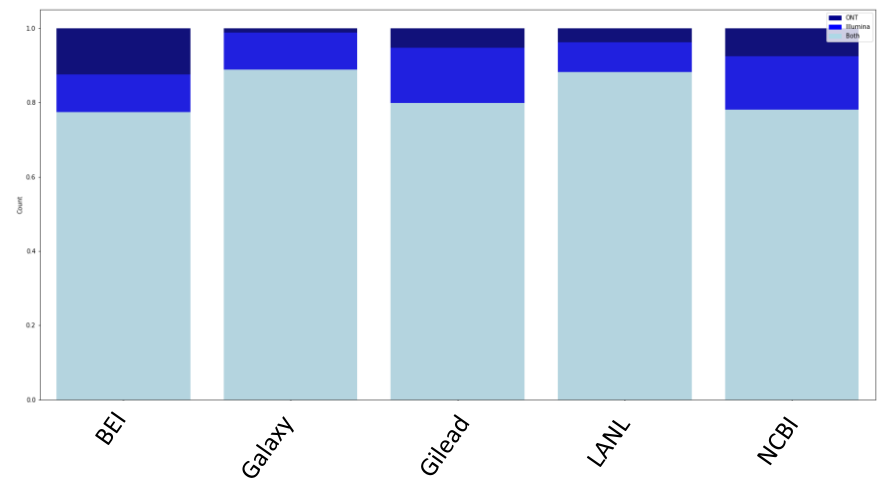

B

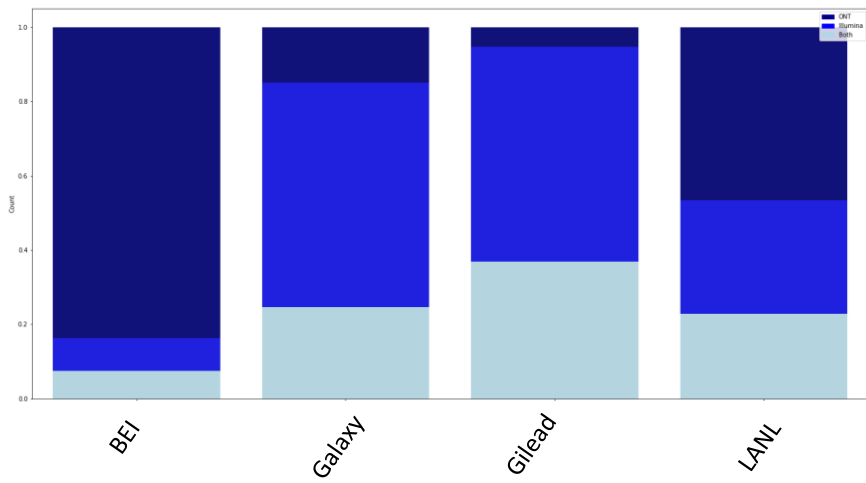

D

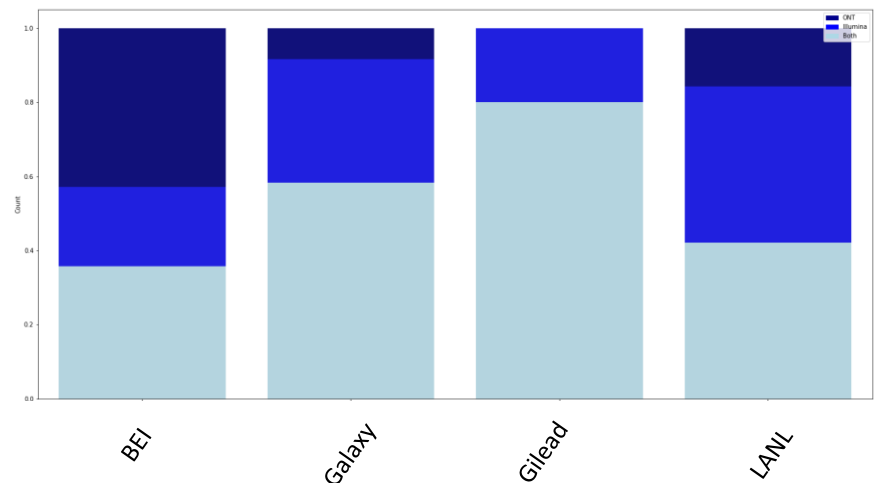

Supplement: Supplement 5 — Supplemental Figure 4. Agreement across platforms with and without recommended parameters, recent dataset. A+B) Agreement between platforms without recommended parameters. C+D) Agreement between platforms with recommended parameters. A+C) Agreement between platforms on SNP calls. B+D) Agreement between platforms on InDel calls. For each figure, only those sample for which both Illumina and ONT platform data had at least one variant call that passed all the filters was considered. The total height is normalized to the total number of calls made by each pipeline, with the light blue portion indicating calls made on both platforms for a given sample, the medium blue indicating calls made only for the Illumina data, and the dark blue indicating calls made only for the ONT data. [file media-5.pdf]
